# Telehealth: improving maternity services by modern technology

**DOI:** 10.1136/bmjoq-2019-000895

**Published:** 2020-11-03

**Authors:** Nusrat Fazal, Anne Webb, Jo Bangoura, Mohamed El Nasharty

**Affiliations:** 1 Obstetrics and Gynecology, Great Western Hospitals NHS Foundation Trust, Swindon, UK; 2 OBGYN, Sidra Medical and Research Center, Doha, Ad Dawhah, Qatar; 3 West of England Academic Health Science Network, Bristol, UK; 4 Department of Obstetrics and Gynaecology, Cairo University, Giza, Egypt

**Keywords:** telemedicine, healthcare quality improvement, obstetrics and gynecology, patient satisfaction, ambulatory care

## Abstract

Hypertension is considered one of the most common medical disorders causing complexities in pregnancy. It could be a newly developed pregnancy-induced hypertension (PIH) or a pre-existing hypertension developing into superimposed pre-eclamptic toxaemia. PIH affects approximately 10% of pregnancies and can have a serious impact on both maternal and fetal well-being; hence requires frequent monitoring and timely intervention. National Institute for Health and Care Excellence (NICE) guidelines recommends once or twice weekly monitoring of blood pressure for such patients. The required frequent monitoring comes with certain implications for patients and healthcare services. An average patient with PIH would need to see her healthcare provider once or twice a week until delivery and 6 weeks thereafter. This certainly increases pressure on limited National Health Service (NHS) resources. Home-based monitoring using Telehealth technology can represent a potential solution for achieving good-quality care for the patient without increasing the workload for healthcare providers. We used ‘Florence’, a text-based technology platform to support home monitoring. We tested its acceptability, feasibility and safety to replace face-to-face appointments for blood pressure monitoring in selected patients with PIH. We implemented our project in three progressive phases using a plan, do, study, act methodology. Florence, telehealth technology was used for blood pressure monitoring instead of face-to-face appointments, and the effect of this innovative technology on the services and the patient experience was studied and necessary modifications were made before progression into the next phase. We recruited 75 patients over 12 months through the progressive phases and replaced around 800 face-to-face appointments by remotely supervised monitoring sessions with Florence successfully, with improved care and patient satisfaction. We also achieved better compliance with the NICE guidelines for blood pressure monitoring in PIH. Our project concluded that Telehealth can be a potential solution for improving care in maternity services, with lesser burden on NHS resources.

## Introduction

Pregnancy-induced hypertension (PIH) complicates 6%–10% of pregnancies.^1^ PIH is defined as new-onset hypertension with or without proteinuria after 20 weeks of gestation. It can lead to serious maternal and fetal consequences like pre-eclampsia, placental abruption, cerebrovascular haemorrhage, liver and renal failure. It can also lead to prematurity, intrauterine growth restriction and fetal death.^2^

The National Institute for Health and Care Excellence (NICE) guideline recommends once weekly monitoring for patients with mild hypertension (those with blood pressure (BP) equal or more than 140/90) and twice weekly for patients with moderate hypertension (those with BP equal or more than 150/100).^3^ This can lead to an excess burden on National Health Service (NHS) resources.^4^

We assessed ourselves against these standards of vigilant monitoring in a busy district hospital with 4800 deliveries per year. Having more the ten thousand attendances to the maternity day assessment unit (DAU), we estimated that 22% of these were purely for BP monitoring. These were in addition to what would have been done in the community by midwives and general practitioners and yet, we could not fully comply with the NICE recommendations. It was estimated that the number of visits to the maternity DAU would have increased by more than 50% to achieve compliance with NICE guidelines recommendations which would cause a further burden on NHS maternity services.

From a patients’ perspective, this frequent hospital-based monitoring would not be feasible either. Coming to the DAU for BP measurement requires taking time off work, arranging for childcare, paying for parking and waiting in antenatal clinic reception until a bed is free. It was estimated that the average time spent at the hospital was up to 2 hours (excluding journey time).

We established a case for change to find an innovative way of monitoring BP in selective patients to reduce the workload on maternity services and improve the patient experience without compromising safety and incurring additional cost for the NHS.

People have been trying to find smarter ways of monitoring chronic conditions at home to reduce frequent visits to healthcare facilities.^5^ Telehealth is one of these tools used for managing chronic hypertension remotely. It is thought to be associated with a reduction in hospital visits, better control of BP and more patient satisfaction.^6^

We wanted to introduce this concept of home monitoring using easily available, affordable and user-friendly telehealth technology for our women with PIH.

We chose the ‘Florence’, a remote monitoring technology, that was piloted in other clinical settings and communicated in regional conferences.^7^

Florence is a simple text-based telehealth system^8 9^ that sends simple text messages to the patients on their mobile phones (does not require smartphones) that send prompts according to preset protocols. For example, it will ask to measure BP, check urine dipstick for proteins or ask general well-being questions. Patients monitor their BP and text the readings back to Florence. These readings are then available to the clinician through a URL programme on any internet device such as smartphones, tablets, laptops or desktops. The data are then reviewed by the clinician and appropriate actions are taken. It also sends automated alerts to clinicians in case of deranged readings requiring prompt actions.^10^

## Specific aims

We aimed to introduce a home-based monitoring system for pregnant women with hypertension, using telehealth technology, to improve the efficiency of maternity services, and patient satisfaction. The objective was to test this new system on at least 50 patients and achieve 80% patient satisfaction with it. We were hoping to see a 10% reduction in face-to-face attendance at healthcare services for BP monitoring. We wanted to assess the acceptability, feasibility and safety of the new system.

## Methods and interventions

**Figure 1:**
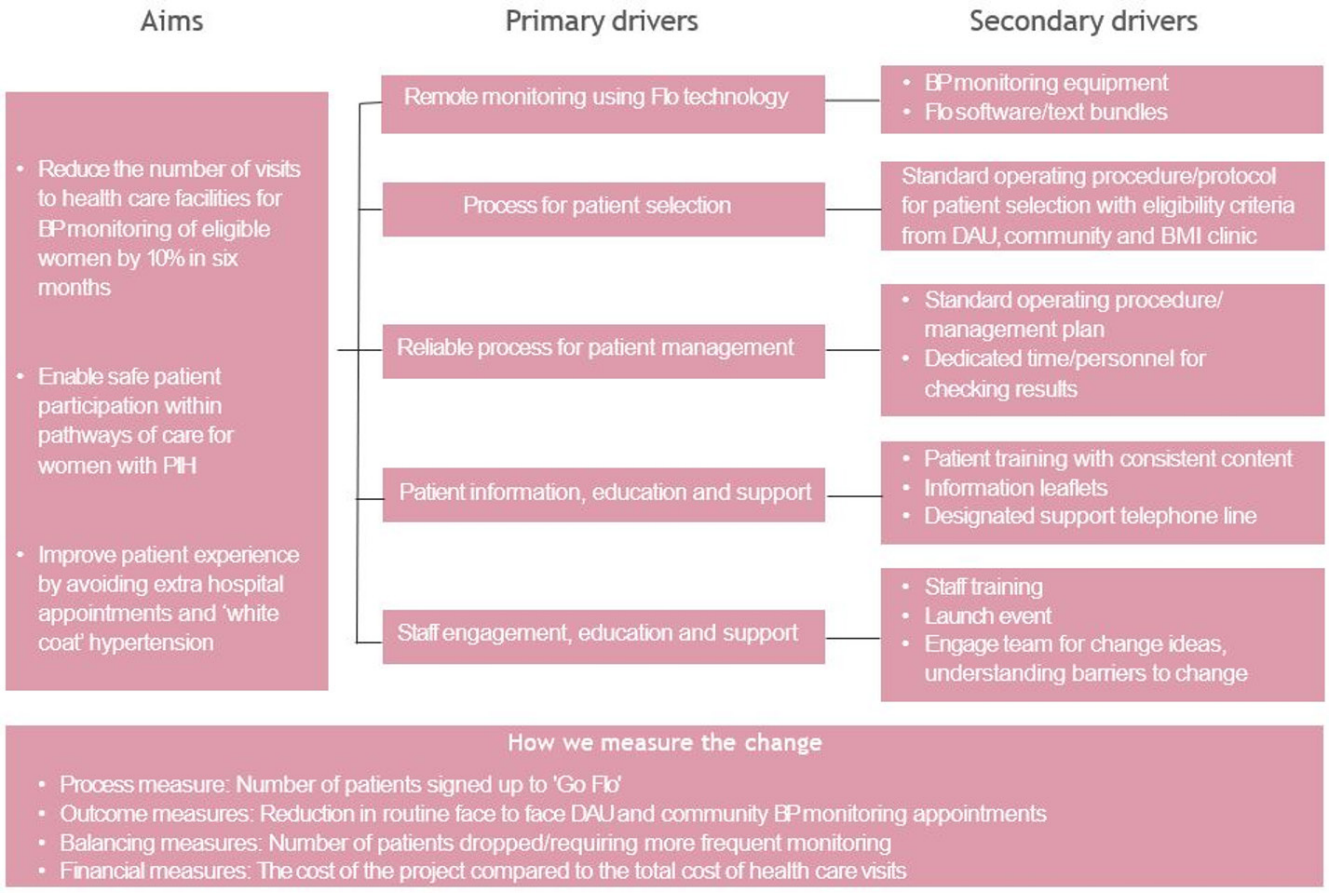
Driver diagram: showing primary and secondary drivers needed to achieve proposed aims. BMI, body mass index; BP, blood pressure; DAU, day assessment unit; PIH, pregnancy induced hypertension.

We drew up an initial driver diagram (a model for improvement) to identify our primary and secondary drivers to start working towards our aims (see figure 1).^11^

A project team was created, consisted of two champions (a clinician and a midwife), a project lead to manage, troubleshoot and communicate with key stakeholders, and a project evaluator to evaluate success and sustainability.

A preagreed protocol for home monitoring of BP was produced. The project was named ‘Go Flo Fridays’. This was to align with the availability of champions on this specific day of the week for consistency in approach. Patients were assessed for eligibility based on fixed selection criteria (online supplemental appendix A).

10.1136/bmjoq-2019-000895.supp1 Supplementary data

Suitable patients, who agreed to participate in the study, were provided with a calibrated BP machine and urine dipstick kits. They were trained through a face-to-face educational session by the champion midwife.

Florence telehealth technology was used to create a dialogue with the patient via text message.^8^ It would remind her every Friday at a set time to check her BP, dipstick urine and send readings back through text. It would also enquire about warning symptoms of pre-eclampsia and fetal movements. Florence would then analyse the response according to preset ranges and triggers. It would either reassure if readings were within the target range or advise to go to a healthcare facility for face-to-face assessment if stated otherwise. DAU staff would review the patient sent responses through Florence URL for all patients every Friday afternoon to ensure safety and take actions accordingly.^12^We used a phased implementation strategy to maintain patients’ safety and followed the plan, do, study, act principles of improvement methodology as we went along depending on feedback from patients and staff.^13^ Patients were recruited in three phases (table 1).

**Table 1:** Planned phased interventions and expected outcomes

Phased implementation	Intervention	Patient selection	Expected outcome
Phase I (2 months)	BP home monitoring without Flo (reported via phone) (an alternated week with F2F initially)	Borderline PIH or risk factors for PIH/PET requiring weekly monitoring	Patient acceptability Patient satisfaction F2F appointment saved Lessons learnt
Phase II (6 months)	BP home monitoring with ‘Flo’ till delivery (weekly)	Mild PIH with risk factors No medication	F2F appointments saved patient satisfaction, system safety operational use. Lessons learnt
Phase III (4–6 months till the time of evaluation)	BP home monitoring with’ Flo’ till delivery (Twice weekly)	Moderate hypertension with medication (single drug)	F2F appointments saved Patient satisfaction System safety Lesson learnt

BP, blood pressure; F2F, face to face; PET, pre-eclampsia toxaemia; PIH, pregnancy induced hypertension.

Phase 1, the pilot phase was run for 2 months on pregnant women with borderline high BP or high risk for pre-eclampsia. The main aim was to study patients’ understanding of the system, acceptability/feasibility of the proposed programme and obtain their feedback to launch the next phase accordingly.u

Phase 2 (implementation phase), involved patients with mild hypertension, not on medications (antihypertensives). They were recruited between 20 weeks and 37 weeks+6 days of gestation, BP less than 150/100, no proteinuria, normal blood results, normal fetal growth, patients with a previous history of pre-eclampsia toxaemia (PET)/PIH not on antihypertensives. (online supplemental appendix A) (online supplemental appendix B) (online supplemental file 3).

10.1136/bmjoq-2019-000895.supp2 Supplementary data

10.1136/bmjoq-2019-000895.supp3 Supplementary data

The patient survey was used to assess patient satisfaction After ensuring patients’ safety, satisfaction and making small changes according to patient /staff feedback, we proceeded to phase 3. This involved patients with moderate PIH, or essential hypertension controlled with medications. Patients were required to be asymptomatic for pre-eclampsia and without fetal complications. Patients in this phase were monitored twice weekly as per NICE guidelines and were contacted for face-to-face appointments if BP was above 150/100, any degree of proteinuria, concerns about fetal movements, or any symptoms of pre-eclampsia. They would be physically contacted if they failed to respond to ensure safety and compliance.

Data were manually collected from dedicated folders maintained for each patient on the DAU by the champion midwife. This included records of any telephonic conversations, issues arising, deranged readings, taking print out of Florence monitoring, any actions are taken, and feedback questionnaires (table 1).

A total of 75 patients were recruited by the end of 12 months between July 2017 and June 2018. Patient and staff satisfaction questionnaires were sent to those who completed the trial.

Our main strategy was to progress slowly and safely to allow modifications as we go along. This was achieved by fortnightly team meetings to study every step and identify areas for improvement. We modified until plans were executed safely and successfully.

We anticipated a reasonable amount of resistance to change, as would be the case for any transformational project, and had strategies in place to deal with it. The lead clinician adopted Deming’s philosophy and approached from different angles.^14 15^

Financial aspects were tackled by engaging the right stakeholders for funding. The lead consultant prepared a business case to estimate the projected cost and saving for NHS resources. psychological factors were taken care of by addressing the team’s anxieties/concerns, providing regular support and evidence of good practice. Safety was ensured by regular staff training, patient education and weekly system reviews. Ethical aspects were addressed by following the Trust’s internal governance processes for approval of a quality improvement project.

The project was regularly monitored and formally evaluated by the Academic Health Science Network to have a formal evaluation report that could be submitted to NHS England for consideration/recommendation to other healthcare facilities.

### Measures

Our main outcome measures were patient satisfaction and reduction in face-to-face visits to healthcare facilities (DAU, community midwife/GP).

The number of appointments saved was estimated by counting the total monitoring episodes covered by ‘Florence’ (according to the NICE guidelines), that would have otherwise required face-to-face appointments at healthcare facilities.

We included balancing measures to capture any adverse events. It was done by noting the number of cases that had to leave the study because of developing complication, deemed not suitable for remote monitoring and to identify issues in the process itself that can impact patient experience or safety.

Financial cost saving was estimated as a secondary outcome. It was calculated using the approximate local finance tariffs per appointment and per hour cost of midwifery time.

## Results

**Figure 2:**
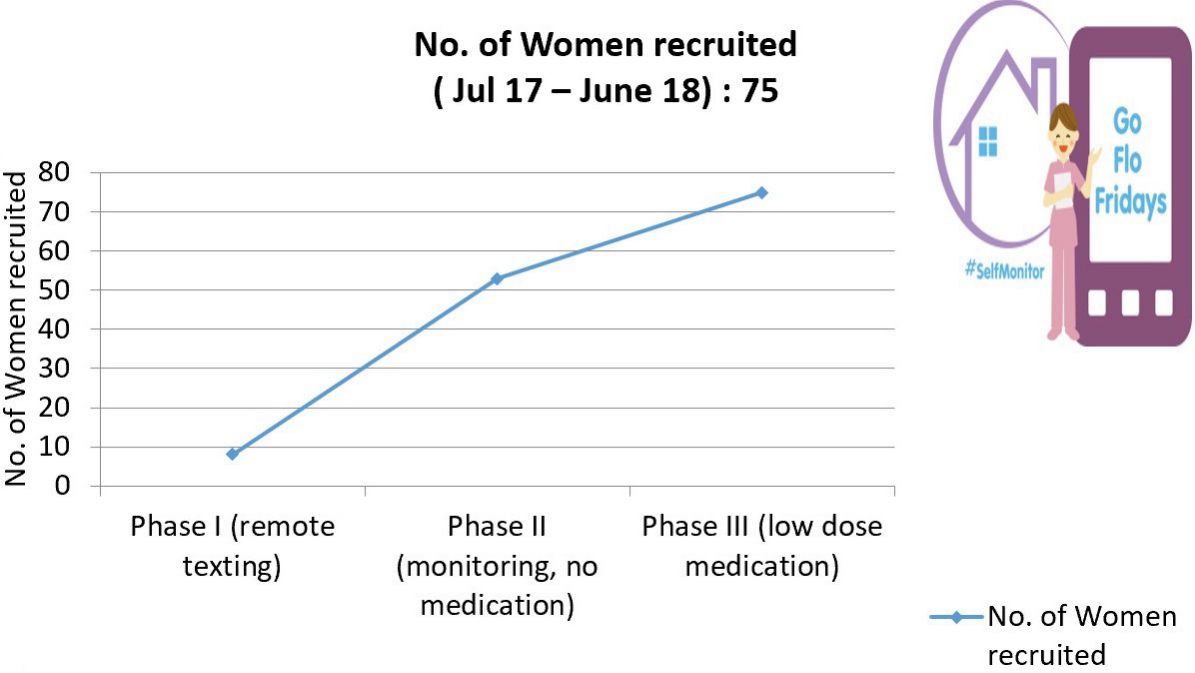
Flo (Florence) uptake/ no of patient signed up to different phases.

A total of 75 women were recruited to the ‘Go Flo’ Project for self-monitoring. Out of which, 8 patients tested the concept in phase 1, 53 used Florence in phase 2 and 14 patients who were on medications joined in phase 3 (figure 2).

Fifty-six women had a chance to complete the trial until delivery. The rest were still using the system (not delivered yet) and had not completed the survey at the time of data collection. Three patients were dropped halfway through the trail due to the development of comorbidities, two of this developed severe pre-eclampsia and one diagnosed with diabetes and were moved to a conventional face-to-face monitoring system.

### Patient satisfaction

Ninety per cent of the patients recruited to phase one found measuring their BP at home acceptable and feasible.

Out of the 56 women who successfully continued with Florence until delivery, 35 returned the completed survey and all of them rated their experience with self-monitoring and Florence as ‘good’ or ‘excellent’. 100% confirmed the recommendation of Florence to friends and family.

Around 14 individual comments were received that were mostly positive. One woman commented that home monitoring would probably be stressful for a first-time mum (online supplemental appendix B). Few patient feedback comments are as follows (table 2).

**Table 2:** Patient feedback comments

Patients	Quotes
Patient A	‘The ‘Go Flo’ has worked really well for me. Being 45 mins away has saved me unnecessary trips to the hospital. Any queries I’ve had have been promptly dealt with’.
Patient B	‘Being able to monitor my BP at home was fantastic. The support provided was so valuable’.
Patient C	‘Absolutely fantastic idea! Really easy to use and so much more practical to monitor myself at home with a toddler’.

BP, blood pressure.

### Reduction in number of visits to healthcare services (DAU/community)

More than 800 healthcare appointments were avoided during the 12 months, out of which 179 reflected reduction in DAU visits and the rest replaced community appointments as mild cases with no medications would have been seen in the community due to limited capacity in DAU. Use of Florence technology potentially saved midwifery time and cost of NHS appointments (table 3).

**Table 3:** Number of appointments replaced by flo (Florence) and potential estimated savings

	DAU and community: appointments saved	DAU and community: hours saved*	Savings as midwifery time (£18.7/hr approx)	Savings as hospital appointments (£70/appt approx)
Phase I	23	23–46	£430–£860	£1610
Phase II	599	599–1198	£11 201–£22 402	£41 930
Phase III	179	179–358	£3347–£6695	£12 530
Total saving	801	801–1602	£14 978–£29 957	£56 070

*Each appointment was estimated to take between 1 and 2 hours of midwifery time from clerking to discharge: including waiting for bed/couch space to be available, 3 BP readings 15 min apart (NICE recommends 30 min apart).

BP, blood pressure; DAU, day assessment unit; NICE, National Institute for Health and Care Excellence.

No major adverse events seen throughout all phases. Two patients were identified with pre-eclampsia and hence moved to face-to-face protocol.

We learnt and modified as we moved forward, for instance, we had to make small adjustments according to staff feedback in phases 2 and 3. We alternated planned Flo monitoring with face-to-face for the first couple of weeks before relying on Florence completely. For example, in phase 3, every Friday our patients measured their BP at home using Florence while we saw them in DAU every Tuesday. This allowed us time for fine-tuning/calibrations of the provided equipment to ensure the accuracy and reliability of the self-monitoring and the system itself.

There were a couple of cases where giving BP equipment rather increased the workload for midwives. One of them was a first-time mum and very anxious. The patient started monitoring every day and kept calling DAU with anxiety. We addressed this problem by educating our patients and discouraging monitoring outside specific set time/day of the week. They were advised to present for face-to-face appointments outside the set days should they have any concerns or develop symptoms, to avoid false reassurance by a BP reading only.

Since the process was assessed and re-assessed, every time improvements were made. For example, we noticed from the survey that one woman would have liked the text codes to be different for different symptoms, so we added a distinct question for symptoms

Some midwives raised concerns about losing the opportunity of asking about symptoms and fetal movements/monitoring and hence questions about PET symptoms and fetal movements were added to the protocol.

Further on in the project, we noticed a system error. An alert was not activated on the BP range of 145/95 but was picked by the midwife reviewing results as a failsafe mechanism. The system provider was contacted immediately and the cut-off for alert was made stricter to ensure safety.

The system allowed reproducibility, previous records that were automatically saved on the machines, were checked now and then to ensure the authenticity of records and compliance. This facility addressed the issue of clinical governance and data storage on a patient’s electronic records.

Vigilant monitoring and regular review of enrolled cases by champions identified a few cases that deviated from inclusion criteria during the trial. One patient was still on the remote monitoring protocol despite hospital admission for BP stabilisation with one-off medication. As a result, the protocol was reviewed, and recommendations were made to switch Florence monitoring to face-to-face monitoring. This initiative was taken to limit the project to mild/moderate hypertension as per the selection criteria and to maintain safety in cases that required more direct care.

Retrieval of BP machines from women was an issue faced by the project team. The regular team meeting strategy was proved helpful in exchanging ideas and finding solutions. The system provider was involved to add a reminder text at the end of the trial and community midwives are now onboard to ensure collection of equipment on day 10 postnatal visit.

The return of the post-trial questionnaire by patients was not very efficient resulting in difficulty with data collection. We addressed the issue by sending a survey via ‘Florence’ that is, through the text system itself to avoid the hassle of filling a paper proforma and using postal service. Florence now sends evaluation questions 4 weeks after enrolment to check the user’s response towards ease of usability that allows time to identification of issues in time and ensures data collection.

At the end of the project, few women were invited for face-to-face interviews to get direct feedback and seek collaboration on areas for improvement. Some of these interviews attracted the media’s attention when women praised the care provided to them through Florence. It helped them feel relaxed and safe at home when weather conditions were not favourable for travel to the hospital. Some reported feeling in control and able to focus more on their jobs.^16^

Miss A said, ‘Go Florence has had a big impact on my current pregnancy and has taken away a lot of the stress and anxiety I was feeling at the beginning as a result of my BP.’

Miss B also said ‘I have always suffered from white coat syndrome and my blood pressure was constantly high during my last pregnancy. This meant I was on a very high dosage of medication which left me feeling lightheaded and I needed to spend one evening every week up DAU which caused me a lot of anxiety’.

Miss C expressed that ‘Absolutely fantastic idea! really easy to use & so much more practical to monitor myself at home with a toddler’.

There was a cost associated with the Florence system in terms of the license (£5000 per annum at the time) and a one-off purchase of BP monitoring equipment to lend, but it was offset by the saved appointments. Also, dedicated midwifery time was secured to monitor the system in the initial phases. It was envisaged that the time saved from face-to-face appointments will enable midwives to focus on higher-risk patients and will alleviate pressure on already stretched resources.

We shared our findings in local and regional meetings and recommended its use to other maternity units and other chronic health conditions.

## Discussion

This project was carried out to introduce and evaluate an innovative way of monitoring pregnant patients with PIH. Our results demonstrated that these women can be monitored in line with NICE guidance using telehealth technology. It can improve patient experience, reduce workload and secure financial savings once embedded in the healthcare system.

We found some difficulty at the start of the trial to encourage other members of the staff to accept the change in care. We used evidence from old audits that showed poor compliance with NICE guidelines despite using the full capacity of our DAU. We used this as a driver for change. Several presentations were made in departmental meetings to get the team on board. other published studies proving the safety and effectiveness of home-based monitoring^7 17–19^ were discussed, and regular team meetings were held for updates, troubleshooting and fine-tuning. This regular engagement of key stakeholders proved useful in overcoming initial resistance.

We assessed patients’ acceptability, satisfaction and safety. Ninety per cent of patients found measuring their BP at home acceptable, which was above our expected target of 80% as a minimum. This was in agreement with Bostock *et al*,^20^ they also found patients’ acceptability for home BP monitoring. This was later supported by van den Heuvel *et al* too.^5^

As for patients’ satisfaction, 100% of participating eligible women, who completed their trial until delivery, rated an overall experience as ‘good’ or ‘excellent’. All of them supported a recommendation to friends and family. These well aligned with the findings of Cottrell *et al*^21^ who reported patients’ satisfaction with the use of Florence for management of their BP and that Florence helped patients to control their BP at their own pace. They recommended its use for future control of BP and other chronic health problems.

Cappuccio *et al*^22^ concluded that home BP monitoring is associated with better control of BP. This was best demonstrated in our patient population who required interventions in previous pregnancies but avoided the need in the current pregnancy due to more vigilant monitoring in their comfort zone, avoiding the influence of hospital anxiety on BP. In our study, remote monitoring helped to distinguish true hypertension from the anxiety-related rise in BP and prevented unnecessary intervention. Ambulatory monitoring was also preferred by Antza *et al* for the same purpose.^23^

In our study, we did not see any adverse event probably due to regular and convenient monitoring that enabled timely detection of cases developing pre-eclampsia, resulting in timely management as observed by Ganapathy *et al*.^24^

AbuDagga *et al*^25^ reported that BP telemonitoring is feasible, effective and reliable with good patients’ satisfaction. They also mentioned that the use of telemonitoring improves patients’ confidence in their care. Similar trend was found in our study reflected by patients’ comments such as ‘The system providing reassurance during the uncertain time’; ‘convenience of monitoring at home and not having to go into hospital’; ‘better readings at home because less stressful than going into DAU’.

We observed that patients were willing to invest in their BP monitor and stay on the top of their health even after delivery. This gave us the confidence of expanding its role to postpartum women to help with achieving better control of BP in the long term, as supported by the work of McManus *et al*.^26^ Our patients were keen to continue using home BP monitoring even after the project was over.

Although a conventional economical model was not used, a simple cost calculation was performed using local figures obtained from the finance department that estimated a potential cost saving. This might be variable in different institutions, using different tariffs but overall promising trends have been demonstrated by subsequent studies too, such as Xydopoulos *et al*.^27^

Stodart *et al*3^28^ reported concerns about the cost of patient training and equipment but it might be considered as a one-off investment that will yield long-term savings. In our study, we did include a one-off cost of buying equipment, but it was easily outweighed by saving the cost of face-to-face appointments. This cost over 12-month period was less than 10% of the potential cost of face-to-face appointments.

Florence, telehealth technology helped to prevent unnecessary treatment for patients who suffer from anxiety-related hypertension, also known as white coat hypertension. This condition represents 15%–30% of patients with high BP^29^ and it is more common in females. These patients are usually not truly hypertensives but because of their anxiety, each time they present to a healthcare provider, they develop hypertension. We saw live examples of this condition during our project reflected by patient feedback. We successfully prevented unnecessary intervention in women who experienced similar episodes in previous pregnancies that were probably interpreted as true hypertension and medications were started. They were induced before the term for this reason. Use of Florence, remote monitoring technology allowed these women to measure their BP conveniently at home to void anxiety-related rise in BP.

We tried to make the project sustainable in long term by getting the support of public health and health service commissioners. We produced a business case, estimated potential savings, and shared the results of our project with them. We believed the use of home monitoring will empower pregnant women to self-monitor their health condition not only during pregnancy but also help develop a mindset towards a healthy lifestyle and taking responsibility for their health. This attitude indeed will prove beneficial in the prevention of chronic disease in the future.

Our project was presented at several regional and national conferences and patient interviews were arranged and covered by local media,^16^ which had increased the awareness and acceptability. Few units approached for sharing protocols for consideration of rollover and will require a more wider and systematic approach for regional/national dissemination and engagement.

### Strengths and limitations

The main strength of our project was that all patients in the trial were followed up by the same team which ensures a standardised level of care. The recruitment process was conducted by the same team to avoid any selection bias.

The project was limited to a very low-risk population and hence the sample size was small. It did not involve patients with chronic hypertension although this group of patients might benefit from home-based monitoring, due to their high risk of developing pre-eclampsia they were initially excluded from the trial. However, after ensuring the safety of Florence, we are planning to extend the inclusion criteria to involve these patients too. The system, however, is operator dependent and hence requires careful selection of patients to avoid misuse of the system. This could be avoided by proper education and training of users of the equipment. Most of the modern BP machines have got built-in memory that stores previous readings and random checks of these can improve the authenticity of BP readings.

## Conclusion

Our project confirmed that remote monitoring using telehealth technology (Florence) can be used safely and effectively for monitoring of BP in women with PIH. It can help in achieving compliance with the NICE guidelines for the frequency of monitoring without increasing workload on healthcare. It has high patient acceptability and satisfaction. It can take the burden off NHS resources and make them available for managing more complicated health conditions.

Since the successful evaluation of this project and excellent patient feedback, Florence is now used as a business as usual at Great Western Hospital. This modern technology is now proving its true value in COVID-19 Pandemic by providing safer, convenient, familiar, and already tested alternative to face-to-face monitoring as also noted by Niela‐Vilen *et al*^30^). Florence is now used in full capacity serving 45–50 women a week and its role has been expanded to high-risk women too, according to the recent update from the lead midwife at the Great Western Hospital.

Great Western Hospital has benefited greatly in being an early adopter of such technology and this has helped during the COVID-19 pandemic. Regional and national collaboration is needed to expand the role/use of modern technology further to improve patient care both nationally and internationally.
