# Supplementary material for: Telehealth: improving maternity services by modern technology
Source: BMJ Open Qual. 2020 Nov 3;9(4):e000895. doi: 10.1136/bmjoq-2019-000895 (PMC7640525; doi:10.1136/bmjoq-2019-000895)
Supplement: Supplementary data [file bmjoq-2019-000895supp001.pdf]

Telehealth Monitoring Selection Criteria (Appendix A)

Inclusion criteria for the telehealth pilot study:

1. Age 18- 40 years
2. Gestation 20weeks -37weeks+6 days. (can *be overridden by Consultant)*
   It is acknowledged that some women would benefit from early gestation monitoring to alert to escalating hypertension based on past medical or obstetric history. This would be part of Consultant-led plan of care
3. BP less than 150/100
4. No proteinuria
5. Normal blood results
6. Normal fetal growth
7. Normal CTG
8. Previous history of preeclampsia
9. Multiple pregnancies
10. Essential hypertension controlled
11. No PET symptoms

Exclusion criteria

1. 1.Age <18 or > 40 years
2. Gestation > 38 weeks or <24 weeks(can be overridden by consultant)
3. BP> 150/100
4. Proteinuria
5. Abnormal blood results
6. Abnormal fetal growth
7. Abnormal CTG
8. Diabetes
9. Renal disorders
10. Previous history of eclampsia or complicated preeclampsia
11. Previous poor obstetric history
12. Patient anxiety or refusal
