# Supplementary material for: Telehealth: improving maternity services by modern technology
Source: BMJ Open Qual. 2020 Nov 3;9(4):e000895. doi: 10.1136/bmjoq-2019-000895 (PMC7640525; doi:10.1136/bmjoq-2019-000895)
Supplement: Supplementary data [file bmjoq-2019-000895supp002.pdf]

"Go Flo" Patient Post-Trial Survey Comments

"Overall, I had a positive experience with the Go Flo system. It provided peace of mind for my family & me. My only suggestion would be amending the text codes. I would highly recommend it"

"Due to severe white coat hypertension, BP readings were always high in a medical setting. At home, they were normal, enabled me to stay off unnecessary medication/interventions".

"Easy to use. Any symptoms felt, could check BP at home & phone if concerned. Reassurance was given with extra check"

"This service is amazing & worth its weight in gold. Everyone wants a hassle-free day/pregnancy. & not having to come up to the hospital to be monitored. Worked perfectly for me & my family's busy life. Thank you, Flo & the team."

"Provides extra reassurance for me at home especially living a distance away"

"Being able to monitor BP at home was fantastic. The support provided was so valuable."

"Being able to test my BP at home has given a true reading; coming into the hospital would have negatively impacted on this. I knew if I was concerned or needed help, I could phone DAU".

“If I was a first-time mum, would probably find it more stressful recording my own results as wouldn’t be sure what was normal.”

“I have been to DAU so many times in the past few weeks, each time everyone has been reassuring & helpful! It’s made what has been a very uncertain time more bearable. Thank you!”

“Thank you for allowing me to take part, it is an excellent idea”

“Absolutely fantastic idea! Really easy to use and so much more practical to monitor myself at home with a toddler. A great system cannot recommend it highly enough”.

“Thank you for helping me monitor my BP so easily.”

“The Go Flo has worked really well for me. Being 45 mins away has saved me unnecessary trips to GWH [hospital]. Any queries I've had have been promptly answered by DAU. Thank you very much!”

“I would like to thank Chestnut clinic for allowing me to use Go Flo. This has taken my concerns away during the pregnancy & using the machine & reminders is great. Thank you again”

“All the midwives were very nice, friendly, and helpful. Was feeling very comfortable and taken care of. Thank you for taking care & looking after me & my baby”
