# Supplementary material for: Telehealth: improving maternity services by modern technology
Source: BMJ Open Qual. 2020 Nov 3;9(4):e000895. doi: 10.1136/bmjoq-2019-000895 (PMC7640525; doi:10.1136/bmjoq-2019-000895)
Supplement: Supplementary data [file bmjoq-2019-000895supp003.pdf]

**Abbreviations:**

BP: Blood Pressure

FLO: Florence

Jul: July

NICE: National Institute of Clinical Excellence

PIH: pregnancy-induced hypertension

PET: pre-eclamptic toxemia

F2F: Face to face

DAU: Day assessment unit
